# Socioeconomic status as an effect modifier of alcohol consumption and harm: analysis of linked cohort data

**DOI:** 10.1016/S2468-2667(17)30078-6

**Published:** 2017-05-10

**Authors:** Srinivasa Vittal Katikireddi, Elise Whitley, Jim Lewsey, Linsay Gray, Alastair H Leyland

**Affiliations:** a Medical Research Council (MRC)/Scottish Government Chief Scientist Office (CSO) Social and Public Health Sciences Unit, University of Glasgow, Glasgow, UK; b Health Economics and Health Technology Assessment, University of Glasgow, Glasgow, UK

## Abstract

**Background:**

Alcohol-related mortality and morbidity are high in socioeconomically disadvantaged populations compared with individuals from advantaged areas. It is unclear if this increased harm reflects differences in alcohol consumption between these socioeconomic groups, reverse causation (ie, downward social selection for high-risk drinkers), or a greater risk of harm in individuals of low socioeconomic status compared with those of higher status after similar consumption. We aimed to investigate whether the harmful effects of alcohol differ by socioeconomic status, accounting for alcohol consumption and other health-related factors.

**Methods:**

The Scottish Health Surveys are record-linked cross-sectional surveys representative of the adult population of Scotland. We obtained baseline demographics and data for alcohol consumption (units per week and binge drinking) from Scottish Health Surveys done in 1995, 1998, 2003, 2008, 2009, 2010, 2011, and 2012. We matched these data to records for deaths, admissions, and prescriptions. The primary outcome was alcohol-attributable admission or death. The relation between alcohol-attributable harm and socioeconomic status was investigated for four measures (education level, social class, household income, and area-based deprivation) using Cox proportional hazards models. The potential for alcohol consumption and other risk factors (including smoking and body-mass index [BMI]) mediating social patterning was explored in separate regression models. Reverse causation was tested by comparing change in area deprivation over time.

**Findings:**

50 236 participants (21 777 men and 28 459 women) were included in the analytical sample, with 429 986 person-years of follow-up. Low socioeconomic status was associated consistently with strikingly raised alcohol-attributable harms, including after adjustment for weekly consumption, binge drinking, BMI, and smoking. Evidence was noted of effect modification; for example, relative to light drinkers living in advantaged areas, the risk of alcohol-attributable admission or death for excessive drinkers was increased (hazard ratio 6·12, 95% CI 4·45–8·41 in advantaged areas; and 10·22, 7·73–13·53 in deprived areas). We found little support for reverse causation.

**Interpretation:**

Disadvantaged social groups have greater alcohol-attributable harms compared with individuals from advantaged areas for given levels of alcohol consumption, even after accounting for different drinking patterns, obesity, and smoking status at the individual level.

**Funding:**

Medical Research Council, NHS Research Scotland, Scottish Government Chief Scientist Office.

## Introduction

Alcohol consumption accounts for a growing burden of death and disability worldwide.^1^ Both alcohol-related deaths (for which alcohol is a contributory cause) and alcohol-attributable deaths (for which alcohol is solely the cause) occur more commonly among disadvantaged socioeconomic groups compared with populations from advantaged areas, thereby contributing to health inequalities.^2^, ^3^, ^4^ However, the mechanisms through which alcohol consumption leads to health inequalities are poorly understood.^5^ Although alcohol-attributable harms are recorded more frequently in disadvantaged socioeconomic groups, gradients in consumption are small or even absent internationally—a finding usually referred to as the alcohol harm paradox.^6^ This situation is the case within Scotland too, where alcohol-related admissions are more than seven times more frequent among people living in the most deprived areas compared with those living in the least disadvantaged areas.^7^ However, few consumption differences have been noted in population-representative survey data.^8^

Several potential explanations exist for the apparent discrepancy between levels of alcohol consumption and rates of alcohol-attributable harms recorded across social groups.^5^ First, methodological differences in how consumption and harms are calculated could be important. Second, differences in the patterns of alcohol consumption—eg, augmented binge drinking—or other known risk factors, including body-mass index (BMI), could be relevant. Third, there is potential for reverse causation, with alcohol consumption resulting in adverse socioeconomic circumstances—ie, downward social selection. Finally, it is possible that the relation between alcohol consumption and subsequent harms could differ between social groups at the individual level.

**Research in context**

**Evidence before this study**

We initially searched MEDLINE and Embase in March, 2013, using keywords including “alcohol”, “socioeconomic status”, “socioeconomic position”, “deprivation”, and “inequalities”. Previous studies suggested a potential increased risk of harm for similar alcohol consumption levels among socioeconomically disadvantaged populations, but whether this hypothesis is correct remains unclear. A systematic review published in 2015 on the relation between socioeconomic status, alcohol consumption, and alcohol-attributable harms concluded that studies investigating the interaction between alcohol-attributable disease, socioeconomic status, and alcohol use are scarce.

**Added value of this study**

To the best of our knowledge, our study is the largest to date investigating whether socioeconomic status effect modifies the relation between alcohol consumption and harm, and is the only one to investigate a range of potential alternative explanations, including reverse causation. Using high-quality survey data linked to admissions, mortality, and community prescriptions, we eliminated several important biases as potential explanations for the higher burden of alcohol-attributable harms in more socioeconomically disadvantaged populations. Alcohol consumption and binge drinking did not differ substantially with socioeconomic status. Despite this finding, the risk of harm was increased strikingly among socioeconomically disadvantaged populations.

**Implications of all the available evidence**

Alcohol-attributable harms are a major contributor to health inequalities. The body of available evidence indicates that this differential burden does not arise simply as a result of higher risk consumption among socioeconomically disadvantaged groups. Efforts to target alcohol consumption by socioeconomic status are unlikely to be successful in reducing health inequalities, unless drinking cultures in the most disadvantaged populations differ systematically from societal norms. Interventions seeking to reduce consumption across the whole population are more likely to result in greater reductions in absolute health inequalities than previously thought. Further research is needed to investigate the reasons for the noted effect modification.

Developing the most effective health policy to narrow inequalities requires an understanding of the alcohol harm paradox. We aimed to assess whether the harmful effects of alcohol consumption differ by socioeconomic status and, if so, the extent to which differences are accounted for by differences in drinking patterns, smoking, and BMI at the individual level.

## Methods

### Data sources

The Scottish Health Surveys are repeated, record-linked, cross-sectional surveys, intended to be representative of the community-dwelling adult population of Scotland.^9^ Trained interviewers survey adults face to face to obtain demographics and lifestyle factors, and they measure height and weight. For our study, we used baseline data from surveys done in 1995, 1998, 2003, 2008, 2009, 2010, 2011, and 2012. During the survey, participants were asked to give informed consent for their data to be linked confidentially to administrative health records, and for data sharing, with at least 85% of participants consenting every year.^10^

### Procedures

We used probabilistic linkage to link Scottish Health Survey data to NHS Scotland's Community Health Index, a unique identifier available for more than 99% of the adult population. We then used the identifier for deterministic linkage to mortality, general inpatient and psychiatry hospital discharge records (respectively, the Scottish Morbidity Records 01 and 04),^9^ and community prescriptions. Validated hospital discharge data were available from 1981 onwards, whereas community prescriptions data were available from 2008. Prescription data for participants who had a prescription filled by a community pharmacist in Scotland also provided follow-up measures of area-based deprivation.

We pooled data for the eight cohorts formed through record linkage, with time at risk starting from the approximate date (month and year) of participation. We excluded participants who had been admitted because of an alcohol-attributable condition before survey baseline measures; for analyses that included binge drinking (for which data were available from 1998 onwards), we excluded the 1995 survey data. Moreover, we excluded participants who had ever been admitted for a drug-related reason or had received a prescription for a medication related to drug dependence—ie, naltrexone, methadone, buprenorphine, and lofexidine. The appendix (p 1) provides details of participants who were excluded. Sensitivity analyses with these participants included did not materially change the findings (data not shown).

Detailed questions about alcohol consumption have been asked consistently across all survey waves (except for binge drinking, which was not included in the 1995 survey) and have been described previously.^11^ We used two key measures of alcohol consumption: number of units consumed in the previous week (with a unit defined as 8 g of pure alcohol); and binge drinking. We classified weekly alcohol consumption into five groups: never drinker or ex-drinker; light drinker (1–10 units for men, 1–7 units for women); moderate drinker (11–20 units for men, 8–13 units for women); heavy drinker (21–50 units for men, 14–35 units for women); and excessive drinker (≥51 units for men, ≥36 units for women). We defined binge drinking on the basis of exceeding the UK Government's recommendations (during the period of data collection) for consumption on 1 day—ie, 6 units for women and 8 units for men.^12^

We investigated multiple dimensions of socioeconomic status, reflecting different stages of the life course and including individual, household, and area-level indicators. We categorised highest educational qualifications into six groups on the basis of the International Standard Classification of Education (ISCED):^13^ degree or above (ISCED levels 6–8); higher national certificate (HNC) or higher national diploma (HND; ISCED level 5); Scottish higher grade or equivalent (ISCED level 4); Scottish standard grade or equivalent (ISCED level 3); other school (ISCED levels 1 or 2); none (ISCED level 0). We categorised social class into six groups on the basis of the Registrar General's classification:^14^ I (professional); II (intermediate); IIINM (skilled non-manual); IIIM (skilled manual); IV (partly skilled); and V (unskilled). We grouped equivalised household income into quintiles accounting for differences in household composition (on a scale of 1–5, with 5 the highest and 1 the lowest). The Scottish Index of Multiple Deprivation (SIMD) is an area-based measure of deprivation that ranks small geographical areas (with a median population of 750 people) on the basis of multiple facets of deprivation identified in administrative data.^15^ SIMD quintiles, therefore, provided an area-based categorical measure of deprivation allowing investigation of social mobility over time (on a scale of 1–5, with 5 the least deprived and 1 the most deprived).

To investigate effect modification, we collapsed each of the socioeconomic status variables into dichotomous high and low categories, so that roughly equal numbers of events occurred within each group. We split highest educational qualification into none, other school, and Scottish standard grade (low) versus Scottish higher grade, HNC, HND, and degree or above (high). We dichotomised area-based deprivation into the most deprived two quintiles (1 and 2; low) versus the least deprived three quintiles (3–5; high). We categorised social class as manual (IIIM, IV, and V; low) versus non-manual (I, II, and IIINM; high) occupations. Finally, we split income into the lowest two quintiles (1 and 2; low) versus the highest three quintiles (3–5; high).

We defined all covariates on the basis of a preplanned analysis protocol and chose them to minimise potential confounding on the basis of previous knowledge. We included age and sex in all statistical models. Further covariates included BMI (derived from interviewer-measured weight and height) and self-reported smoking status, which we categorised into five groups: never smoker, ex-smoker, current light smoker (fewer than ten cigarettes per day), current moderate smoker (ten to 19 cigarettes per day), and current heavy smoker (20 or more cigarettes per day).

We also assessed the potential for reverse causation—ie, downward social selection occurring as a result of high-risk alcohol consumption. We compared baseline area-based deprivation as assessed in the Scottish Health Surveys (measured in quintiles) with that ascertained during follow-up (identified by flagging the last available deprivation quintile from postcode in prescriptions, admissions, and mortality data).

### Outcomes

The primary outcome was either alcohol-attributable admission or alcohol-attributable death, which we defined on the basis of standardised International Classification of Diseases ninth revision (ICD-9) and tenth revision (ICD-10) diagnostic codes,^16^, ^17^ with the list of conditions in the appendix (p 2). To limit disclosure risk, we used approximate dates (month and year). Mortality and hospital discharge data in Scotland have been shown previously to be more than 99% complete and have more than 90% accuracy.^18^, ^19^

The secondary outcome was any of alcohol-attributable death, alcohol-attributable admission, or prescription for a medication deemed to be related to alcohol dependence (namely, acamprosate, disulfiram, and chlordiazepoxide). We did sensitivity analyses to allow for the addition of diazepam and thiamine as alcohol-related medications.

### Statistical analysis

For the primary analysis, we used Cox proportional hazards models to investigate associations between exposures of interest and the first episode of an alcohol-attributable outcome. The proportional hazards assumption was met. In view of the potential non-linearity in associations between alcohol consumption and harms, we fitted a fractional polynomial function for alcohol consumption to allow for the best functional form to be determined.^20^ Our initial model for the association between an alcohol-attributable event and each of the socioeconomic status variables included age (as a fractional polynomial function), sex, and survey wave. We then included in nested models the following covariates, incrementally: alcohol consumption; binge drinking; smoking status; and BMI (modelled as a fractional polynomial). We calculated p values with the Wald test based on combined hazard ratio (HR) and SE estimates obtained by application of Rubin's rules for combining multiply imputed data. We next assessed the potential for associations between consumption and harms to differ by socioeconomic status; we categorised the sample on the basis of both alcohol consumption and socioeconomic status. The reference group for this analysis was light alcohol consumption among individuals with high socioeconomic status, and we compared relative risks against this reference group. We presented graphically the probability of having alcohol-attributable harm for a given alcohol consumption, stratified by socioeconomic status. There was no strong evidence of any statistical interactions with sex and age and we, therefore, present results for all sex and age-groups combined.

Because community prescriptions data were unavailable before 2008, it was not possible to model the secondary outcome using survival analysis. Therefore, we treated the secondary outcome as a binary variable, classifying participants into those who had or did not have the outcome during the period of follow-up, and modelled it using logistic regression. This approach assumed no differential receipt of alcohol-related prescriptions before 2008 and is, therefore, likely to underestimate inequalities. We did sensitivity analyses of logistic regression models in the same manner as for the primary outcome.

We investigated the potential for downward social selection as a result of alcohol consumption through cross-tabulation of SIMD quintiles over time, stratified by baseline weekly alcohol consumption and binge drinking. We then used linear regression models (adjusting for age, sex, and survey wave) to test explicitly the association between high-risk alcohol consumption and social mobility. Because SIMD information at follow-up was only available for a subsample of all participants (72% of the main sample analysed), we did a robustness check of the primary analysis using these data to ensure that differences did not arise as a result of selection bias in the analytical sample. Similarly, we restricted our analysis of social selection to participants from 2008 onwards and excluding ascertainment of deprivation from deaths in robustness analyses.

All variables analysed were more than 88% complete (appendix p 3). However, to minimise the potential for bias arising from missing data, we fitted all statistical models to data with ten rounds of multiple imputation, using chained equations,^21^ with all terms (including interactions) contained within the imputation model. We did all analyses in Stata version 13.2.

### Data sharing

The full dataset is available to researchers via an application to NHS Scotland's Privacy Advisory Committee.

### Role of the funding source

The funders of the study had no role in study design, data collection, data analysis, data interpretation, or writing of the report. The corresponding author had full access to all the data in the study and had final responsibility for the decision to submit for publication.

## Results

50 236 participants (21 777 men and 28 459 women) were included in the analytical sample (table 1). Considerable variation in drinking behaviours was noted by sex, with men tending to pursue high-risk drinking patterns (4236 [19%] men and 3206 [11%] women reported binge drinking in the previous week). Women were more likely to be never smokers and ex-smokers and to have a slightly lower BMI, compared with men. During follow-up of 429 986 person-years, 1022 people achieved the primary outcome—ie, alcohol-attributable death or admission (appendix p 4)—with more men than women affected. Using the broader secondary outcome of alcohol-attributable death, admission, or receipt of a prescription related to alcohol dependence, this number increased to 1398 affected individuals (table 1). The appendix (pp 5–8) summarises alcohol consumption according to the four different measures of socioeconomic status, illustrating relatively comparable consumption across socioeconomic groups in cross-sectional data.

**Table 1:** Characteristics of study sample

		**Men (n=21 777)**	**Women (n=28 459)**	**Total (n=50 236)**
Survey wave				
	1995	3176 (15%)	4002 (14%)	7178 (14%)
	1998	3491 (16%)	4541 (16%)	8032 (16%)
	2003	3165 (15%)	4055 (14%)	7220 (14%)
	2008	2327 (11%)	3056 (11%)	5383 (11%)
	2009	2656 (12%)	3505 (12%)	6161 (12%)
	2010	2552 (12%)	3482 (12%)	6034 (12%)
	2011	2667 (12%)	3573 (13%)	6240 (12%)
	2012	1743 (8%)	2245 (8%)	3988 (8%)
Age at interview (years)		48·3 (17·5)	48·0 (17·5)	48·1 (17·5)
Smoking status*†				
	Never smoker	9345 (43%)	13 414 (47%)	22 759 (45%)
	Ex-smoker	6629 (30%)	7293 (26%)	13 922 (28%)
	Current light smoker	1126 (5%)	1911 (7%)	3037 (6%)
	Current moderate smoker	2208 (10%)	3314 (12%)	5522 (11%)
	Current heavy smoker	2268 (10%)	2403 (8%)	4671 (9%)
Body-mass index (kg/m^2^)		27·4 (4·6)	27·3 (5·7)	27·4 (5·3)
Drinking status*‡				
	Never drinker	766 (4%)	2169 (8%)	2935 (6%)
	Ex-drinker	1153 (5%)	1787 (6%)	2940 (6%)
	Light drinker	8626 (40%)	15 147 (53%)	23 773 (47%)
	Moderate drinker	5033 (23%)	4716 (17%)	9749 (19%)
	Heavy drinker	4764 (22%)	3798 (13%)	8562 (17%)
	Excessive drinker	1257 (6%)	643 (2%)	1900 (4%)
Binge drinking in past week*				
	Never or ex-drinker	1919 (9%)	3956 (14%)	5875 (12%)
	No binge drinking	11 307 (52%)	15 110 (53%)	26 417 (53%)
	Binge drinking§	4236 (19%)	3206 (11%)	7442 (15%)
Total person-years		186 123·7	243 862·5	429 986·2
Alcohol admissions and deaths		655 (3%)	367 (1%)	1022 (2%)
Alcohol admissions and deaths, or prescriptions		817 (4%)	581 (2%)	1398 (3%)

Data are number of participants (%) or mean (SD).

* Numbers do not sum to total because of missing values.

† Light smoker defined as fewer than ten cigarettes per day, moderate smoker as ten to 19 cigarettes per day, and current heavy smoker as 20 or more cigarettes per day.

‡ Light drinker defined as 1–10 units per week for men, 1–7 units per week for women; moderate drinker as 11–20 units for men, 8–13 units for women; heavy drinker as 21–50 units for men, 14–35 units for women; and excessive drinker as ≥51 units for men, ≥36 units for women.

§ Defined as >6 units per day for women and >8 units per day for men.

In unadjusted analyses, and after adjustment for age, sex, and survey wave, alcohol-attributable events occurred more frequently among socially disadvantaged groups, according to all four socioeconomic status measures (table 2). Associations recorded were large, with more than three-fold higher rates of alcohol-attributable harms among the most disadvantaged populations compared with the most advantaged, according to all four socioeconomic status measures. Adjustment for alcohol consumption and binge drinking had little effect on the magnitude of the associations seen. Further adjustment for BMI and smoking status attenuated associations only slightly, suggesting that the social patterning of alcohol-attributable harms might not be accounted for by differences in these other risk factors. For example, the HR for participants with no qualifications compared with people educated to degree level was 3·76 (95% CI 2·96–4·77) when adjusting for age, sex and survey wave; additional adjustment for weekly alcohol consumption and binge drinking left the risk relatively unchanged (3·44, 2·61–4·52), whereas further adjustment for smoking and BMI attenuated the HR to 2·50 (1·88–3·31). Analysis of the secondary outcome, which uses a broader definition of alcohol-attributable harm and includes prescription for a medication related to alcohol dependence, yielded similar results (appendix pp 9–12).

**Table 2:** Risk of alcohol-attributable admission or death according to socioeconomic status (multiply imputed data)

		**Events/person-years**	**Adjusted for age, sex, and survey wave**		**Adjusted for age, sex, survey wave, alcohol consumption, and binge drinking**		**Adjusted for age, sex, survey wave, alcohol consumption, binge drinking, BMI, and smoking status**	
			HR (95% CI)	p*	HR (95% CI)	p*	HR (95% CI)	p*
Highest educational qualification†		..	..	<0·0001	..	<0·0001	..	<0·0001
	Degree or above (ISCED 6–8)	85/80 693·6	1·00	..	1·00	..	1·00	..
	HNC or HND (ISCED 5)	82/45 934·5	1·62 (1·20–2·20)	..	1·38 (0·96–1·99)	..	1·24 (0·86–1·79)	..
	Scottish higher grade (ISCED 4)	89/52 234·2	1·69 (1·26–2·28)	..	1·63 (1·15–2·31)	..	1·51 (1·06–2·14)	..
	Scottish standard grade (ISCED 3)	296/111 984·5	2·55 (2·00–3·26)	..	2·29 (1·72–3·05)	..	1·86 (1·39–2·49)	..
	Other school (ISCED 1 or 2)	51/20 408·8	2·77 (1·94–3·94)	..	2·52 (1·69–3·77)	..	1·99 (1·32–2·98)	..
	None (ISCED 0)	419/118 070·2	3·76 (2·96–4·77)	..	3·44 (2·61–4·52)	..	2·50 (1·88–3·31)	..
Area-based deprivation (quintiles)		..	..	<0·0001	..	<0·0001	..	<0·0001
	5 (least deprived)	93/76 272·8	1·00	..	1·00	..	1·00	..
	4	142/86 077·9	1·36 (1·05–1·77)	..	1·37 (0·98–1·91)	..	1·27 (0·91–1·77)	..
	3	166/87 729·6	1·58 (1·23–2·04)	..	1·75 (1·28–2·40)	..	1·51 (1·10–2·07)	..
	2	243/89 746·8	2·32 (1·83–2·95)	..	2·62 (1·94–3·54)	..	2·11 (1·56–2·86)	..
	1 (most deprived)	377/89 099·5	3·66 (2·92–4·59)	..	3·72 (2·79–4·98)	..	2·71 (2·01–3·64)	..
Social class		..	..	..	..	..	..	..
	I (professional)	21/18 562·3	1·00	<0·0001	1·00	<0·0001	1·00	<0·0001
	II (intermediate)	153/108 653·9	1·50 (0·95–2·37)	..	1·27 (0·76–2·12)	..	1·18 (0·70–1·92)	..
	IIINM (skilled non-manual)	132/94 416·3	1·93 (1·21–3·07)	..	1·51 (0·89–2·58)	..	1·30 (0·76–2·21)	..
	IIIM (skilled manual)	262/81 515·1	2·69 (1·72–4·20)	..	2·14 (1·29–3·53)	..	1·65 (0·99–2·73)	..
	IV (partly skilled)	255/71 712·5	4·01 (2·57–6·27)	..	3·37 (2·04–5·57)	..	2·44 (1·47–4·05)	..
	V (unskilled)	129/29 536·7	5·22 (3·28–8·30)	..	4·33 (2·54–7·38)	..	3·02 (1·76–5·17)	..
Income (quintile)		..	..	<0·0001	..	<0·0001	..	<0·0001
	5 (highest)	41/35 541·1	1·00	..	1·00	..	1·00	..
	4	56/33 306·9	1·51 (1·01–2·26)	..	1·58 (1·04–2·39)	..	1·46 (0·96–2·22)	..
	3	72/31 373·3	2·17 (1·47–3·19)	..	2·30 (1·55–3·43)	..	1·99 (1·33–2·97)	..
	2	80/33 254·4	2·36 (1·61–3·47)	..	2·83 (1·91–4·20)	..	2·24 (1·50–3·34)	..
	1 (lowest)	110/25 150·6	4·41 (3·07–6·33)	..	4·85 (3·32–7·09)	..	3·58 (2·43–5·27)	..

BMI=body-mass index. HNC=Higher National Certificate. HND=Higher National Diploma. HR=hazard ratio.

* p values are for the linear trend across socioeconomic status categories, calculated with the Wald test based on combined HR and SE estimates obtained by the application of Rubin's rules.

† Defined with the International Standard Classification of Education (ISCED).^13^

**Figure 1:**
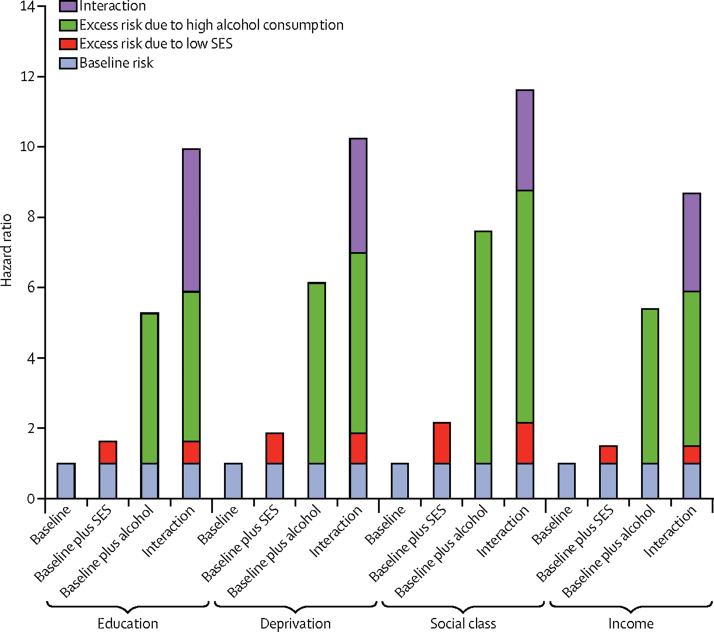
Risks of alcohol-attributable harms by alcohol consumption and socioeconomic status Adjusted for age, sex, study wave, smoking, body-mass index, and binge drinking in the past week. SES=socioeconomic status.

**Figure 2:**
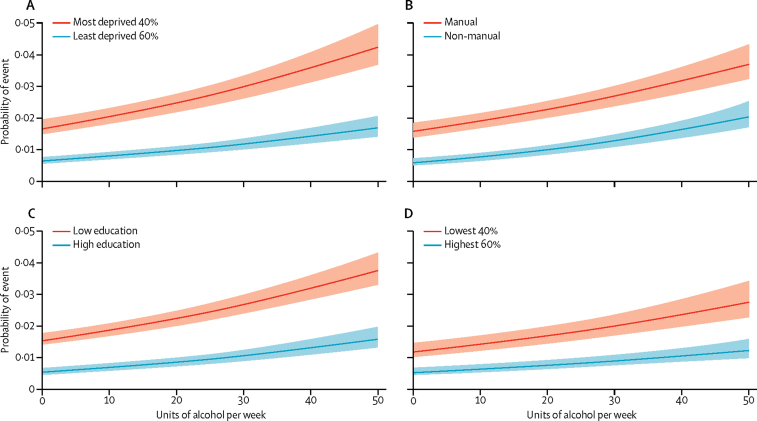
Predicted probability of an alcohol-attributable event during follow-up, stratified by socioeconomic status All models were adjusted for age, sex, study wave, smoking, body-mass index, and binge drinking in the past week. The lines represent the predicted probability of experiencing an alcohol-attributable event (estimated from logistic regression models) and the shading denotes the 95% CI. (A) Deprivation was categorised according to the most deprived two quintiles *vs* the least deprived three quintiles. (B) Social class categorisation was based on manual *vs* non-manual occupations. (C) Education was categorised according to attainment of none, other school, or Scottish standard grade qualifications *vs* Scottish higher grade, higher national certificate, higher national diploma, or degree or above. (D) Household income was categorised by the lowest two quintiles *vs* highest three quintiles. Follow-up for household income measures was shorter because participants were not asked about this information in the first two study waves of data collection.

Effect modification was noted for all four socioeconomic status measures on alcohol consumption for alcohol-attributable harms (table 3, figure 1). For example, compared with light drinkers living in areas of low deprivation, the HR for excessive drinkers in similar areas was 6·12 (95% CI 4·45–8·41) but rose to 10·22 (7·73–13·53) for excessive drinkers living in deprived areas. To ease interpretation, figure 2 presents equivalent results from the logistic regression analyses graphically. Lower socioeconomic groups had a greater absolute risk of harm for any given level of consumption but additionally, increased consumption is generally associated with disproportionately increased risk of alcohol-attributable harm among those of lower socioeconomic status. This relation was recorded across all four measures, but was least evident when assessed by social class. For completeness, we checked for and did not find effect modification on a multiplicative scale (appendix pp 13, 14).

**Table 3:** Risk of alcohol-attributable admission or death according to drinking status, by socioeconomic status (multiply imputed data)

		**High socioeconomic status**		**Low socioeconomic status**	
		Events/person-years	HR (95% CI)*	Events/person-years	HR (95% CI)*
Highest educational qualification†					
	Never or ex-drinker	7/11 698·3	0·76 (0·35–1·65)	49/32 542·4	1·50 (1·03–2·19)
	Light drinker	63/80 831·6	1·00 (ref)	203/126 202·8	1·62 (1·22–2·17)
	Moderate drinker	50/41 951·7	1·35 (0·93–1·96)	153/45 262·5	2·95 (2·19–3·98)
	Heavy drinker	87/36 814·7	2·42 (1·75–3·36)	218/36 101·3	4·77 (3·58–6·36)
	Excessive drinker	33/6656·6	5·26 (3·56–7·77)	132/8537·1	9·92 (7·27–13·54)
By area-based deprivation‡					
	Never or ex-drinker	22/20 973·8	1·18 (0·74–1·87)	34/23 206·4	1·39 (0·94–2·05)
	Light drinker	103/119 676·7	1·00 (ref)	163/87 277·7	1·85 (1·44–2·37)
	Moderate drinker	73/54 588·6	1·40 (1·04–1·91)	130/32 487·0	3·46 (2·66–4·50)
	Heavy drinker	130/45 090·2	2·77 (2·13–3·60)	174/27 707·3	5·05 (3·93–6·48)
	Excessive drinker	64/8387·2	6·12 (4·45–8·41)	112/6798·9	10·22 (7·73–13·53)
By occupational class§					
	Never or ex-drinker	10/17 862·5	0·76 (0·39–1·46)	35/21 656·7	1·67 (1·11–2·50)
	Light drinker	76/109 125·4	1·00 (ref)	172/86 639·2	2·16 (1·64–2·84)
	Moderate drinker	58/48 320·2	1·62 (1·15–2·28)	134/34 855·9	3·76 (2·81–5·02)
	Heavy drinker	109/39 095·6	3·52 (2·62–4·73)	179/30 725·0	5·37 (4·05–7·11)
	Excessive drinker	49/6492·7	7·59 (5·26–10·94)	119/7910·4	11·60 (8·54–15·76)
By income¶					
	Never or ex-drinker	5/7817·9	0·57 (0·23–1·44)	14/10 543·2	1·05 (0·57–1·91)
	Light drinker	49/45 393·6	1·00 (ref)	58/30 082·4	1·50 (1·02–2·21)
	Moderate drinker	28/22 230·7	1·09 (0·68–1·73)	29/8664·1	2·24 (1·40–3·56)
	Heavy drinker	51/20 319·4	1·98 (1·33–2·93)	58/6584·3	5·36 (3·62–7·93)
	Excessive drinker	34/3770·8	5·39 (3·44–8·44)	31/1892·9	8·67 (5·45–13·79)

Light drinker defined as 1–10 units per week for men, 1–7 units per week for women; moderate drinker as 11–20 units for men, 8–13 units for women; heavy drinker as 21–50 units for men, 14–35 units for women; and excessive drinker as ≥51 units for men, ≥36 units for women. BMI=body-mass index. HNC=Higher National Certificate. HND=Higher National Diploma. HR=hazard ratio.

* Adjusted for age, sex, survey wave, smoking, BMI, and binge drinking in past week.

† None, other school, Scottish standard grade (low socioeconomic status) *vs* Scottish higher grade, HNC, HND, degree or above (high socioeconomic status).

‡ Most deprived two quintiles *vs* least deprived three quintiles.

§ Manual *vs* non-manual occupations.

¶ Lowest two quintiles *vs* highest three quintiles.

To investigate the extent to which social selection might contribute to the social patterning of alcohol-attributable harms, SIMD quintile membership was compared between survey year and latest follow-up with reference to alcohol consumption reported in the survey. Most participants did not change deprivation category but the most movement was noted among participants consuming the greatest volume of alcohol (table 4) and who engaged in binge drinking (appendix pp 15–20), although this change included both upward and downwards mobility. When examined in regression analyses, higher baseline alcohol consumption and binge drinking were both associated with only very slight upward social mobility.

**Table 4:** Difference in area-based deprivation quintile between baseline and follow-up (from first admission, prescription, or death), by drinking status at baseline

		**Never or ex-drinker (n=3629)**	**Light or moderate drinker (n=18** **439)**	**Heavy or excessive drinker (n=5351)**
Negative difference		175 (5%)	1249 (7%)	400 (7%)
	–4 (biggest downward difference)	9 (<1%)	46 (<1%)	16 (<1%)
	–3	14 (<1%)	144 (1%)	45 (1%)
	–2	41 (1%)	326 (2%)	125 (2%)
	–1	111 (3%)	733 (4%)	214 (4%)
No difference (0)		3270 (90%)	15 915 (86%)	4492 (84%)
Positive difference		184 (5%)	1275 (7%)	459 (9%)
	1	118 (3%)	692 (4%)	251 (5%)
	2	41 (1%)	352 (2%)	123 (2%)
	3	16 (<1%)	193 (1%)	65 (1%)
	4 (biggest upward difference)	9 (<1%)	38 (<1%)	20 (<1%)
Mean difference relative to non-drinkers (95% CI)				
	Adjusted for baseline deprivation quintile	0·00	0·05 (0·02–0·07)	0·07 (0·04–0·10)
	Adjusted for baseline deprivation quintile, age, and sex	0·00	0·04 (0·02–0·07)	0·06 (0·03–0·09)
	Adjusted for baseline deprivation quintile, age, sex, and time from interview to prescription	0·00	0·04 (0·02–0·07)	0·06 (0·03–0·09)
	Adjusted for baseline deprivation quintile, age, sex, time from interview to prescription, and wave	0·00	0·04 (0·02–0·07)	0·06 (0·03–0·09)

Data are number of participants (%), unless otherwise indicated.

Robustness analyses for the secondary outcome (appendix pp 13, 14), complete cases (appendix pp 15–20), restricting the analysis to the 72% subsample with longitudinal deprivation data (appendix pp 21, 22), and alternative approaches to investigating social selection (appendix pp 23–29) all yielded similar findings to the main results.

## Discussion

Our findings show that alcohol-attributable harms are far higher in disadvantaged social groups, even when accounting for differences in consumption and binge drinking and irrespective of which measure of socioeconomic status is used. These inequalities are not accounted for by differences in smoking or BMI. Increased alcohol consumption is associated with harm in all socioeconomic groups, but disproportionately so for individuals with low socioeconomic status. There is little evidence for reverse causation—that is, high-risk consumption leading to social disadvantage is not the explanation for these findings.

Our study has several strengths. By contrast with most previous studies, we used data linkage to ensure that differences in the sampling frames for people reporting consumption and those experiencing harm did not result in selection bias and we overcame the possible ecological fallacy by using individual-level data. Availability of high-quality administrative data allowed us to exclude participants who had previously had alcohol-attributable harms, thereby minimising potential reverse causation. Furthermore, we investigated the issue of reverse causation directly. We studied several dimensions of socioeconomic status and reported remarkably consistent results across measures. The robustness of our findings was assessed using several prespecified outcomes.

Some limitations of our study should be noted. First, although the Scottish Health Surveys are intended to be representative of the general population, they systematically under-represent some groups at higher risk of harm,^10^, ^22^ and a small proportion of the cohort were excluded because of non-consent for linkage. Therefore, our study might not allow inferences to be made about the most severely disadvantaged populations, such as homeless people. Second, our study relies on self-reported alcohol consumption. Theoretically, systematic reporting differences between social groups could result in bias, although our use of several measures of socioeconomic status reduces the likelihood of this bias. Furthermore, existing research suggests that differential reporting between sociodemographic groups does not seem to be an important source of bias.^23^ Third, we have only been able to investigate the effect of total weekly alcohol consumption and binge drinking and not more detailed patterns of alcohol consumption and different types of drink. Similarly, our consumption measures were taken at one timepoint, rather than capturing information on lifetime drinking. The finding of greater alcohol-attributable harms among very light drinkers with a low socioeconomic status could reflect previous consumption patterns. This possibility could be the case among the combined category of never drinkers and ex-drinkers (who were pooled for statistical power), because ex-drinkers include people who stop consuming alcohol because of the development of health conditions or previous dependence. The consistency in findings across socioeconomic measures—which reflect different points in the life course and the magnitude of social patterning of harms that persisted after adjustment—make these factors less likely to account for our overall findings. However, if high-risk drinkers with low socioeconomic status are less likely to modify their drinking than are people with high socioeconomic status after the survey, this could also result in unaccounted time-varying confounding. Fourth, systematic differences in ascertainment of outcomes could be possible if some socioeconomic groups were less likely to be identified through administrative records. This risk is minimised by the very low prevalence of private health care provision in Scotland and the high ascertainment of outcomes possible through linkage to the Community Health Index, which allows for emigration from Scotland to be identified.

In a previous meta-analysis of longitudinal research, an increased risk of alcohol-attributable harms was noted among more disadvantaged social groups.^24^ McDonald and colleagues^25^ previously studied the relation between alcohol consumption and alcohol-attributable harms, also using linked Scottish Health Survey data, and they noted higher harms with area-level deprivation. Similarly, Makela and colleagues^2^ reported greater harms in manual (compared with non-manual) workers using linked Finnish survey data. In a cross-sectional study in New Zealand,^26^ self-reported measures of alcohol-related harms did not differ between social groups once patterns of consumption were accounted for. Although the relation between socioeconomic status and alcohol-attributable and alcohol-related harms is well established, there is little consensus about the mechanisms underpinning this relation. Evidence about whether high-risk alcohol consumption is more common among disadvantaged communities is mixed, with some studies describing greater levels of consumption among more socioeconomically advantaged individuals,^27^ but greater binge drinking,^28^ and more extreme drinking among those more disadvantaged.^29^ In keeping with our results, findings of a study in England showed evidence of the alcohol harm paradox, irrespective of which measure of socioeconomic status was used.^30^

Reducing social inequalities in health remains a priority. To date, much of the alcohol-orientated policy effort in many countries has been focused on reducing alcohol consumption among more disadvantaged social groups.^31^ Our study findings show that the risk of alcohol-attributable harms among moderate alcohol consumers of low socioeconomic status is greater than for those who drink heavily but are socioeconomically advantaged. The lived experiences of poverty shape the emergence of health outcomes, not only through health-related behaviours but also as a result of poor material circumstances and psychosocial stresses.^32^ Poverty might, therefore, reduce resilience to disease, predisposing drinkers of low socioeconomic status to greater health harms despite exposure to similar levels of risk factors as drinkers of high socioeconomic status.

Our findings highlight the need for policy to prioritise the tackling of inequalities in alcohol-attributable harms. Furthermore, reductions in overall levels of population consumption seem most likely to narrow health inequalities. Findings of modelling studies suggest that the introduction of price-based policy interventions, such as minimum unit pricing, will yield greatest benefits to disadvantaged groups.^33^ Such studies have typically assumed that similar consumption of alcohol results in comparable levels of harm among different social groups. Because similar alcohol consumption exerts greatest harm among more disadvantaged groups, the effect of population-based prevention efforts on health inequalities have the potential to be larger than previously anticipated. This outcome is especially relevant because of the planned implementation of minimum unit pricing in Scotland, which has been delayed substantially after legal challenges from the alcohol industry^34^, ^35^—the beneficial effects in terms of reduced health inequalities could be even greater than currently expected.

Further research to understand the mechanisms by which health inequalities arise is needed. In relation to alcohol-attributable harms, this effort requires investigating relations between consumption patterns by social groups and specific diseases.^36^ In the future, more nuanced measures of alcohol consumption over time and biological measurements of liver function could address current weaknesses in the evidence base. Furthermore, investigating the contribution of detailed nutritional factors and physical activity might suggest other mechanisms amenable to modification. Our study has focused on a narrow definition of harm—quantifying the broader range of alcohol-related harms will reveal the true magnitude of the alcohol harm paradox. Findings of previous studies on other important modifiable risk factors—eg, smoking and obesity—have shown moderately increased harms arising from comparable doses among socially disadvantaged groups.^37^ Future studies are now needed to investigate further modifiable risk factors and better understand the comparative effectiveness of specific interventions, including health and social policies, to narrow inequalities.
