# Supplementary material for: Socioeconomic status as an effect modifier of alcohol consumption and harm: analysis of linked cohort data
Source: Lancet Public Health. 2017 May 10;2(6):e267–76. doi: 10.1016/S2468-2667(17)30078-6 (PMC5463030; doi:10.1016/S2468-2667(17)30078-6)
Supplement: Supplementary appendix [file mmc1.pdf]

THE LANCET
Public Health

Supplementary appendix

This appendix formed part of the original submission and has been peer reviewed. We post it as supplied by the authors.

Supplement to: Katikireddi SV, Whitley E, Lewsey J, Gray L, Leyland AH. Socioeconomic status as an effect modifier of alcohol consumption and harm: analysis of linked cohort data. *Lancet Public Health* 2017; published online May 10. http://dx.doi.org/10.1016/S2468-2667(17)30078-6.

Web Appendix 1: Exclusions by data collection wave

	1995	1998	2003	2008	2009	2010	2011	2012
N respondents	7,932	9,340	11,420	8,170	10,116	9,015	9,510	6,602
N over 16	7,932	9,040	8,107	6,428	7,517	7,229	7,530	4,815
N (%) over 16 refused follow-up	569<br>(7.2)	735<br>(8.1)	662<br>(8.2)	840<br>(13.1)	1,100<br>(14.6)	941<br>(13.0)	1,023<br>(13.6)	674<br>(14.0)
N (%) over 16 with alcohol event pre-interview or ever experiencing a drug-related event	185<br>(2.3)	273<br>(3.0)	225<br>(2.8)	205<br>(3.2)	256<br>(3.4)	254<br>(3.5)	267<br>(3.6)	153<br>(3.2)
N (%) over 16s in analytical sample	7,178<br>(90.5)	8,032<br>(88.9)	7,220<br>(89.1)	5,383<br>(83.7)	6,161<br>(82.0)	6,034<br>(83.5)	6,240<br>(82.9)	3,988<br>(82.8)

In general, the Scottish Health Survey excludes households that have previously participated in the survey from the sampling frame. There were a handful of occasions when the same individual was recruited into the study on two occasions (following the person moving address). When this occurred, baseline data for analysis were chosen randomly.

Web Appendix 2

Alcohol-related hospital episodes

Prior to 01/04/1996

ICD-9 codes hospital episodes	2651, 3039, 3050, 2918, 2910, 2913, 2915, 2919, 2911, 2912, 2918, 3575, 4255, 5353, 5710, 5711, 5712, 5713, 7607, 7598, 7903, 9800, 9801, 9809, E860, V57, E9473, E9509, E9809, E8600*, E8601*, E8609*, E9509* 5711§, 6554§, 2550§, 3594§, 5709§, 3483§, D3039+A3317[a], D3039+A3344[a]

01/04/1996 onward

ICD-10 codes	F10, K70, X45, X65, Y15, Y90, Y91, E244, E512, G312, G621, G721, I426, K292, K860, O354, P043, Q860, R780, T510, T511, T519, Y573, Z502, Z714, Z721

Alcohol-related deaths

Prior to 01/01/2000

Main ICD-9 codes deaths	4255, 5710, 5711, 5712, 5713, 5714, 5715, 5718, 5719, E860, 291, 303, 305

01/01/2000 onward

ICD-10 codes deaths	G312, G621, I426, K292, K740, K741, K742, K746, K860, F10, K70, K73, X45, X65, Y15

The ICD codes here follow those used by Information Services Division NHS National Services Scotland (e.g. http://www.isdscotland.org/Health-Topics/Drugs-and-Alcohol-Misuse/Publications/2014-02-25/2014-02-25-ARHS2012-13-Report.pdf) and the National Records of Scotland (e.g. http://www.gro-scotland.gov.uk/statistics/theme/vital-events/deaths/alcohol-related/coverage-of-stats.html) at the time of data acquisition, for alcohol-related hospital episodes and alcohol-related deaths respectively.
Any pair of an *external alcohol code** and *external code*§ constitutes an alcohol-related episode.
[a] Combination of these codes in consecutive diagnoses listed constitutes an alcohol-related episode.

The protocol for this study is available from: http://www.sphsu.mrc.ac.uk/publications/reports-and-protocols.html

Web Appendix 3: Completeness of key variables for the analytical sample

	N (%) with non-missing data (based on available waves)	Non-availability
Highest educational qualification	50,168 (99.9)	
Deprivation quintile	50,089 (99.7)	
Social class	47,656 (94.9)	
Household income	31,075 (88.7)	Not in 1995/1998
Smoking	49,911 (99.4)	
BMI	44,271 (88.1)	
Current drinking status	49,859 (99.2)	
Binge drinking (among drinkers)	33,859 (89.7)	Not in 1995
Binge drinking (including non-drinkers)	*39,168 (91.0)*	*Not in 1995*

Web Appendix 4: Diagnostic codes responsible for primary outcome events

**Category**	**ICD-9 codes**	**ICD-10 codes**	**No of events**
Mental health and alcohol dependence	2910, 2911, 2912, 2913, 2915, 2918, 2919, 3039, D3039+A3317, D3039+A3344, V57, E9473, *291, 303*	F10, Z50.2, Z71.4, *F10*	672
Neurological	2651, 3575, 3483, 3594, *3575*	G31.2, G62.1, G72.1, *G31.2, G62.1*	5
Liver disease	5709, 5710, 5711, 5712, 5713, *5710, 5711, 5712, 5713*	K70, *K70, K73, K74*	113
Other GI	5353, *5353*	K29.2, K86.0, *K29.2, K86.0*	18
Alcohol detected in blood	7903, *7903*	R78.0, Y90, Y57.3, Y91, Z72.1	97
Alcohol poisoning	3050, 9800, 9801, 9809, E860, E9509, E9809, E8600, E8601, E8609, E9509, *E8600, E8609, 305*	T51.0, T51.1, T51.9, X45, X65, Y15, *X45, X65, Y15*	117
Maternal and foetal alcohol-related outcomes	7607, 7598, 6554	O35.4, P04.3, Q86.0	0
Cardiovascular	4255, *4255*	I42.6, *I42.6*	0
Endocrine conditions	2550	E24.4, E51.2	0

Codes for deaths indicated using italics.

Web Appendix 5: Descriptive tables of alcohol consumption by socioeconomic status

Table 1: Drinking status by highest educational qualification (in those with complete data)

	Highest qualification					
	Degree or higher	HNC/D or equivalent	Higher or equivalent	Standard grade or equivalent	Other school level	None
Current drinking						
Never drinker	477 (4.3)	167 (3.2)	275 (4.2)	507 (4.6)	237 (7.6)	1,266 (10.0)
Ex-drinker	380 (3.4)	231 (4.4)	250 (3.9)	511 (4.6)	290 (9.3)	1,270 (10.1)
Current light drinker	4,924 (43.9)	2,460 (46.5)	3,017 (46.5)	5,386 (48.8)	1,659 (53.1)	6,296 (49.9)
Current moderate drinker	2,675 (23.8)	1,142 (21.6)	1,365 (21.0)	2,083 (18.9)	506 (16.2)	1,970 (15.6)
Current heavy drinker	2,387 (21.3)	1,089 (20.6)	1,278 (19.7)	2,026 (18.4)	354 (11.3)	1,420 (11.3)
Current Excessive drinker	381 (3.4)	202 (3.8)	306 (4.7)	530 (4.8)	79 (2.5)	400 (3.2)
Binge drinker (among drinkers)						
No binge	7,208 (79.9)	2,856 (73.8)	3,513 (73.4)	5,019 (72.4)	1,968 (88.5)	5,811 (83.3)
Binge	1,818 (20.1)	1,013 (26.2)	1,272 (26.6)	1,912 (27.6)	257 (11.6)	1,167 (16.7)
Binge drinker (including non-drinkers)						
Non-drinker	*857 (8.7)*	*398 (9.3)*	*525 (9.9)*	*1,018 (12.8)*	*527 (19.2)*	*2,536 (26.7)*
No binge	*7,208 (72.9)*	*2,856 (66.9)*	*3,513 (66.2)*	*5,019 (63.1)*	*1,968 (71.5)*	*5,811 (61.1)*
Binge	*1,818 (18.4)*	*1,013 (23.7)*	*1,272 (24.0)*	*1,912 (24.1)*	*257 (9.3)*	*1,167 (12.3)*

Table 2: Drinking status by deprivation quintile (in those with complete data)

	Deprivation quintile				
	1 (Least deprived)	2	3	4	5 (Most deprived)
Current drinking					
Never drinker	407 (4.5)	510 (4.9)	545 (5.2)	687 (6.9)	771 (7.8)
Ex-drinker	289 (3.2)	470 (4.5)	625 (6.0)	695 (7.0)	852 (8.6)
Current light drinker	4,018 (44.7)	5,064 (48.4)	5,084 (48.9)	4,925 (49.3)	4,623 (46.7)
Current moderate drinker	2,065 (23.0)	2,171 (20.8)	2,017 (19.4)	1,734 (17.4)	1,735 (17.5)
Current heavy drinker	1,885 (21.0)	1,867 (17.9)	1,745 (16.8)	1,563 (15.7)	1,473 (14.9)
Current Excessive drinker	324 (3.6)	377 (3.6)	380 (3.7)	378 (3.8)	437 (4.4)
Binge drinker (among drinkers)					
No binge	5,370 (80.1)	6,025 (79.5)	5,714 (79.1)	4,882 (77.3)	4,343 (73.2)
Binge	1,331 (19.9)	1,557 (20.5)	1,508 (20.9)	1,437 (22.7)	1,588 (26.8)
Binge drinker (including non-drinkers)					
Non-drinker	*611 (8.4)*	*913 (10.8)*	*1,065 (12.9)*	*1,248 (16.5)*	*1,449 (19.6)*
No binge	*5,370 (73.2)*	*6,025 (70.9)*	*5,714 (69.0)*	*4,882 (64.5)*	*4,343 (58.9)*
Binge	*1,331 (18.2)*	*1,557 (18.3)*	*1,508 (18.2)*	*1,437 (19.0)*	*1,588 (21.5)*

Table 3: Drinking status by social class (in those with complete data)

	Social class					
	I	II	IIINM	IIIM	IV	V
Current drinking						
Never drinker	79 (3.4)	596 (4.4)	625 (5.8)	362 (4.0)	555 (6.6)	290 (9.0)
Ex-drinker	50 (2.2)	559 (4.1)	578 (5.4)	620 (6.8)	659 (7.8)	335 (10.3)
Current light drinker	963 (41.4)	6,248 (46.2)	5,589 (51.7)	3,940 (43.4)	4,204 (49.8)	1,697 (52.4)
Current moderate drinker	624 (26.8)	2,872 (21.2)	2,085 (19.3)	1,915 (21.1)	1,443 (17.1)	456 (14.1)
Current heavy drinker	528 (22.7)	2,740 (21.2)	1,627 (15.1)	1,754 (19.3)	1,248 (14.8)	363 (11.2)
Current Excessive drinker	82 (3.5)	505 (3.7)	302 (2.8)	485 (5.3)	342 (4.1)	100 (3.1)
Binge drinker (among drinkers)						
No binge	1,473 (79.7)	8,092 (79.4)	5,848 (80.1)	4,367 (72.1)	4,233 (77.3)	1,450 (81.0)
Binge	376 (20.3)	2,102 (20.6)	1,451 (19.9)	1,692 (27.9)	1,244 (22.7)	340 (19.0)
Binge drinker (including non-drinkers)						
Non-drinker	*121 (6.1)*	*1,051 (9.4)*	*1,099 (13.1)*	*897 (12.9)*	*1,106 (16.8)*	*562 (23.9)*
No binge	*1,473 (74.8)*	*8.092 (72.0)*	*5,848 (69.6)*	*4,367 (62.8)*	*4,233 (64.3)*	*1,450 (61.7)*
Binge	*376 (19.1)*	*2,102 (18.7)*	*1,451 (17.3)*	*1,692 (24.3)*	*1,244 (18.9)*	*340 (14.5)*

Table 4: Drinking status by household income quintile (in those with complete data)

	Household income quintile				
	1 (Highest)	2	3	4	5 (Lowest)
Current drinking					
Never drinker	183 (2.7)	216 (3.3)	368 (6.0)	491 (7.8)	501 (10.2)
Ex-drinker	186 (2.7)	278 (4.2)	413 (6.7)	558 (8.9)	590 (12.0)
Current light drinker	2,780 (40.3)	3,119 (47.4)	3,042 (49.5)	3,244 (51.7)	2,436 (49.6)
Current moderate drinker	1,712 (24.8)	1,421 (21.6)	1,098 (17.9)	1,025 (16.3)	638 (13.0)
Current heavy drinker	1,712 (24.8)	1,296 (19.7)	1,006 (16.4)	784 (12.5)	535 (10.9)
Current Excessive drinker	319 (4.6)	250 (3.8)	222 (3.6)	176 (2.8)	216 (4.4)
Binge drinker (among drinkers)					
No binge	4,601 (73.0)	4,527 (77.5)	4,037 (79.7)	4,006 (83.4)	2,830 (80.4)
Binge	1,699 (27.0)	1,312 (22.5)	1,030 (20.3)	798 (16.6)	689 (19.6)
Binge drinker (including non-drinkers)					
Non-drinker	*369 (5.5)*	*494 (7.8)*	*781 (13.4)*	*1,049 (17.9)*	*1,091 (23.7)*
No binge	*4,601 (69.0)*	*4,527 (71.5)*	*4,037 (69.0)*	*4,006 (68.4)*	*2,830 (61.4)*
Binge	*1,699 (25.5)*	*1,312 (20.7)*	*1,030 (17.6)*	*798 (13.6)*	*689 (15.0)*

Web Appendix 6: Logistic regression models for secondary outcomes

Table 1: Odds ratio (95% confidence interval) for alcohol admissions/deaths plus prescriptions according to drinking status by socioeconomic status (multiply imputed data)

	Higher socioeconomic position		Lower socioeconomic position	
	n events / Total N	OR (95% CI)[1]	n events / Total N	OR (95% CI)[1]
By highest educational qualification[2]				
Non-drinker	19 / 1,780	1.33 (0.81, 2.18)	82 / 4,081	1.79 (1.32, 2.34)
Light drinker (ref)	93 / 10,401	1.00	297 / 13,341	1.73 (1.36, 2.20)
Moderate drinker	70 / 5,182	1.34 (0.98, 1.84)	199 / 4,559	2.95 (2.29, 3.81)
Heavy drinker	110 / 4,754	2.16 (1.63, 2.87)	277 / 3,800	4.84 (3.79, 6.19)
Excessive drinker	68 / 889	6.15 (4.42, 8.55)	164 / 1,009	10.43 (7.91, 13.76)
By deprivation[3]				
Non-drinker	40 / 2,846	1.50 (1.05, 2.14)	61 / 3,005	1.75 (1.29, 2.38)
Light drinker (ref)	144 / 14,166	1.00	246 / 9,548	2.01 (1.62, 2.48)
Moderate drinker	96 / 6,253	1.37 (2.03, 3.22)	173 / 3,469	3.50 (2.78, 4.41)
Heavy drinker	162 / 5,497	2.56 (2.03, 3.22)	224 / 3,036	5.10 (4.09, 6.36)
Excessive drinker	91 / 1,081	6.75 (5.09, 8.93)	141 / 815	11.06 (8.53, 14.35)
By social class[4]				
Non-drinker	26 / 2,487	1.16 (0.76, 1.78)	62 / 2,821	1.84 (1.35, 2.52)
Light drinker (ref)	125 / 12,800	1.00	245 / 9,841	1.94 (1.56, 2.43)
Moderate drinker	77 / 5,581	1.33 (1.00, 1.78)	177 / 3,814	3.31 (2.60, 4.21)
Heavy drinker	138 / 4,895	2.74 (2.14, 3.51)	225 / 3,365	4.60 (3.64, 5.82)
Excessive drinker	71 / 889	7,06 (5.18, 9.64)	153 / 927	11.03 (8.44, 14.42)

By income[5]				
Non-drinker	15 / 1,644	1.18 (0.67, 2.08)	33 / 2,140	1.75 (1.14, 2.67)
Light drinker (ref)	71 / 8,941	1.00	98 / 5,680	1.83 (1.33, 2.50)
Moderate drinker	43 / 4,231	1.18 (0.80, 1.73)	51 / 1,663	2.91 (2.01, 4.22)
Heavy drinker	74 / 4,014	2.02 (1.45, 2.81)	85 / 1,319	6.00 (4.31, 8.34)
Excessive drinker	52 / 791	6.19 (4.24, 9.02)	58 / 392	12.74 (8.71, 18.65)

[1]Adjusted for age, sex, wave, smoking, BMI and binge drinking in last week; [2]None/other school/standard grade (lower socioeconomic status) versus Higher/HNC/HND/Degree or higher (higher socioeconomic status); [3]Most deprived two quintiles versus least deprived three quintiles; [4]Manual versus non-manual occupations; [5]Lowest two quintiles versus highest three quintiles

Table 2: Odds ratio (95% confidence interval) for alcohol admissions/deaths plus extended alcohol-related prescriptions (including diazepam and thiamine) according to drinking status by socioeconomic position (multiply imputed data)

	Higher socioeconomic position		Lower socioeconomic position	
	n events / Total N	OR (95% CI)[1]	n events / Total N	OR (95% CI)[1]
By highest educational qualification[2]				
Non-drinker	262 / 1,780	1.08 (0.94, 1.25)	874 / 4,081	1.55 (1.40, 1.71)
Light drinker (ref)	1,447 / 10,401	1.00	2,529 / 13,341	1.28 (1.19, 1.38)
Moderate drinker	626 / 5,182	0.89 (0.80, 0.98)	874 / 4,559	1.33 (1.21, 1.46)
Heavy drinker	629 / 4,754	0.95 (0.86, 1.06)	828 / 3,800	1.59 (1.44, 1.76)
Excessive drinker	147 / 889	1.19 (0.98, 1.43)	305 / 1,009	2.47 (2.12, 2.87)
By deprivation[3]				
Non-drinker	477 / 2,846	1.17 (1.05, 1.30)	658 / 3,005	1.44 (1.30, 1.60)
Light drinker (ref)	2,092 / 14,166	1.00	1,889 / 9,548	1.23 (1.15, 1.32)
Moderate drinker	821 / 6,253	0.90 (0.83, 0.99)	679 / 3,469	1.29 (1.17, 1.42)
Heavy drinker	803 / 5,497	1.00 (0.92, 1.10)	653 / 3,036	1.49 (1.35, 1.65)
Excessive drinker	213 / 1,081	1.45 (1.23, 1.70)	239 / 815	2.14 (1.82, 2.52)
By social class[4]				
Non-drinker	411 / 2,487	1.11 (0.99, 1.25)	626 / 2,821	1.53 (1.37, 1.69)
Light drinker (ref)	1,954 / 12,800	1.00	1,840 / 9,841	1.25 (1.16, 1.34)
Moderate drinker	784 / 5,581	0.95 (0.87, 1.04)	664 / 3,814	1.23 (1.12, 1.36)
Heavy drinker	743 / 4,895	1.03 (0.94, 1.13)	656 / 3,365	1.45 (1.30, 1.60)
Excessive drinker	170 / 889	1.31 (1.09, 1.56)	266 / 927	2.35 (2.00, 2.75)
By income[5]				

Non-drinker	282 / 1,644	1.23 (1.07, 1.42)	439 / 2,140	1.44 (1.27, 1.63)
Light drinker (ref)	1,291 / 8,941	1.00	1,077 / 5,680	1.28 (1.17, 1.41)
Moderate drinker	543 / 4,231	0.91 (0.82, 1.02)	289 / 1,663	1.21 (1.05, 1.40)
Heavy drinker	526 / 4,014	0.90 (0.81, 1.01)	287 / 1,319	1.59 (1.37, 1.84)
Excessive drinker	142 / 791	1.28 (1.06, 1.56)	125 / 392	2.56 (2.04, 3.22)

[1]Adjusted for age, sex, wave, smoking, BMI and binge drinking in last week; [2]None/other school/standard grade (lower socioeconomic status) versus Higher/HNC/HND/Degree or higher (higher socioeconomic status); [3]Most deprived two quintiles versus least deprived three quintiles; [4]Manual versus non-manual occupations; [5]Lowest two quintiles versus highest three quintiles

Web Appendix 7: Assessing effect modification on a multiplicative scale

Hazard ratio (95% confidence interval) for alcohol admissions/deaths according to drinking status by socioeconomic position (multiply imputed data)

	Lower socioeconomic position		Higher socioeconomic position		***p*[1]**
	N events / person years	HR (95% CI)[2]	N events / person years	HR (95% CI)[2]	
By highest educational qualification[3]					
Non-drinker	49 / 32,542.4	0.94 (0.68, 1.28)	7 / 11,698.3	0.72 (0.33, 1.57)	
Light drinker (ref)	203 / 126,202.8	1.00	63 / 80,831.6	1.00	
Moderate drinker	153 / 45,262.5	1.78 (1.44, 2.20)	50 / 41,951.7	1.43 (0.99, 2.08)	
Heavy drinker	218 / 36,101.3	2.86 (2.34, 3.48)	87 / 36,814.7	2.53 (1.82, 3.53)	
Excessive drinker	132 / 8,537.1	5.92 (4.69, 7.48)	33 / 6,656.6	5.28 (3.52, 7.93)	
P[4]		*<0.001*		*<0.001*	***0.98***
By deprivation[5]					
Non-drinker	34 / 23,206.4	0.76 (0.52, 1.10)	22 / 20,973.8	1.16 (0.73, 1.84)	
Light drinker (ref)	163 / 87,277.7	1.00	103 / 119,676.7	1.00	
Moderate drinker	130 / 32,487.0	1.85 (1.47, 2.34)	73 / 54,588.6	1.43 (1.05, 1.93)	
Heavy drinker	174 / 27,707.3	2.69 (2.15, 3.37)	130 / 45,090.2	2.79 (2.14, 3.64)	
Excessive drinker	112 / 6,798.9	5.50 (4.25, 7.11)	64 / 8,387.2	6.08 (4.38, 8.44)	
P[4]		*<0.001*		*<0.001*	***0.42***

By occupational class[6]					
Non-drinker	35 / 21,656.7	0.77 (0.53, 1.11)	10 / 17,862.5	0.76 (0.39, 1.47)	
Light drinker (ref)	172 / 86,639.2	1.00	76 / 109,125.4	1.00	
Moderate drinker	134 / 34,855.9	1.71 (1.36, 2.15)	58 / 48,320.2	1.65 (1.17, 2.33)	
Heavy drinker	179 / 30,725.0	2.45 (1.97, 3.05)	109 / 39,095.6	3.56 (2.63, 4.80)	
Excessive drinker	119 / 7,910.4	5.33 (4.15, 6.84)	49 / 6,492.7	7.39 (5.06, 10.79)	
P[4]		*<0.001*		*<0.001*	***0.20***
By income[7]					
Non-drinker	14 / 10,543.2	0.73 (0.41, 1.31)	5 / 7,817.9	0.50 (0.20, 1.27)	
Light drinker (ref)	58 / 30,082.4	1.00	49 / 45.393.6	1.00	
Moderate drinker	29 / 8,664.1	1.42 (0.90, 2.23)	28 / 22,230.7	1.14 (0.71, 1.83)	
Heavy drinker	58 / 6,584.3	3.39 (2.33, 4.93)	51 / 20.319.4	2.12 (1.42, 3.17)	
Excessive drinker	31 / 1,892.9	5.38 (3.41, 8.48)	34 / 3,770.8	5.85 (3.68, 9.29)	
P[4]		*<0.001*		*<0.001*	***0.45***

[1]p for interaction between lower and higher socioeconomic position from case-complete model; [2]Adjusted for age, sex, wave, smoking, BMI and binge drinking in last week; [3]None/other school/standard grade (lower socioeconomic position) versus Higher/HNC/HND/Degree or higher (higher socioeconomic position; [4]p for linear trend across drinking categories; [5]Most deprived two quintiles versus least deprived three quintiles; [6]Manual versus non-manual occupations; [7]Lowest two quintiles versus highest three quintiles

Web Appendix 8: Complete case analyses

Table 1: Hazard ratio (95% confidence interval) for alcohol admissions according to socioeconomic position (sample limited to complete cases)

	N events / person years	Adjusted for age, sex and data collection wave	Adjusted for age, sex, wave, alcohol consumption and binge drinking	Adjusted for age, sex, wave, alcohol, binge drinking, BMI and smoking status
Highest educational qualification				
None	203 / 54,820.2	1.00	1.00	1.00
Other school	35 / 12,398.8	0.74 (0.42, 1.30)	0.82 (0.57, 1.18)	0.90 (0.62, 1.30)
Standard grade	137 / 49,327.4	0.94 (0.63, 1.43)	0.67 (0.53, 0.84)	0.76 (0.60, 0.96)
Higher	54 / 27,800.2	0.54 (0.33, 0.91)	0.51 (0.37, 0.70)	0.66 (0.48, 0.90)
HNC/HND	42 / 25,336.4	0.50 (0.28, 0.91)	0.39 (0.28, 0.55)	0.49 (0.34, 0.69)
Degree or higher	55 / 50,958.5	0.34 (0.21, 0.54)	0.27 (0.20, 0.37)	0.38 (0.28, 0.52)
p[1]		*<0.001*	*<0.001*	*<0.001*
Deprivation				
1 Most deprived quintile	180 / 40,743.2	1.00	1.00	1.00
2	137 / 43,275.9	0.70 (0.56, 0.88)	0.71 (0.57, 0.89)	0.79 (0.64, 0.99)
3	91 / 46,688.9	0.43 (0.33, 0.55)	0.44 (0.34, 0.57)	0.53 (0.41, 0.68)
4	68 / 47,366.9	0.31 (0.24, 0.41)	0.33 (0.25, 0.43)	0.42 (0.31, 0.56)
5 Least deprived quintile	50 / 42,273.9	0.25 (0.19, 0.35)	0.26 (0.19, 0.36)	0.37 (0.26, 0.50)
p[1]		*<0.001*	*<0.001*	*<0.001*

Social class				
V (unskilled)	63 / 13,370.3	1.00	1.00	1.00
IV (partly skilled)	140 / 36,077.0	0.78 (0.58, 1.05)	0.74 (0.55, 1.00)	0.76 (0.56, 1.03)
IIIM (skilled manual)	129 / 40,866.5	0.46 (0.34, 0.63)	0.44 (0.32, 0.60)	0.49 (0.36, 0.66)
IIINM (skilled non-manual)	68 / 47,602.0	0.35 (0.25, 0.50)	0.34 (0.24, 0.49)	0.42 (0.30, 0.60)
II (Intermediate)	90 / 62,442.3	0.28 (0.20, 0.38)	0.25 (0.18, 0.35)	0.33 (0.24, 0.47)
I (Professional)	15 / 11,097.9	0.22 (0.12, 0.38)	0.22 (0.12, 0.39)	0.31 (0.18, 0.56)
p[1]		*<0.001*	*<0.001*	*<0.001*
Income				
1 Lowest quintile	83 / 18,074.4	1.00	1.00	1.00
2	65 / 24.838.5	0.55 (0.40, 0.77)	0.56 (0.40, 0.78)	0.60 (0.43, 0.84)
3	56 / 24.138.0	0.47 (0.33, 0.66)	0.46 (0.32, 0.64)	0.54 (0.38, 0.76)
4	45 / 27,369.8	0.32 (0.22, 0.46)	0.31 (0.21, 0.44)	0.39 (0.27, 0.56)
5 Highest quintile	30 / 29,377.4	0.19 (0.12, 0.29)	0.17 (0.11, 0.26)	0.24 (0.15, 0.36)
p[1]		*<0.001*	*<0.001*	*<0.001*

[1]p for linear trend across socioeconomic status categories

Table 2: Odds ratio (95% confidence interval) for alcohol admissions, deaths and/or prescriptions according to socioeconomic position (sample limited to complete cases)

	n (%) events / N	Adjusted for age, sex and data collection wave	Adjusted for age, sex, wave, alcohol consumption and binge drinking	Adjusted for age, sex, wave, alcohol, binge drinking, BMI and smoking status
Highest educational qualification				
None	290 (3.7) / 7,782	1.00	1.00	1.00
Other school	51 (2.2) / 2,293	0.77 (0.56, 1.04)	0.74 (0.54, 1.01)	0.82 (0.60, 1.13)
Standard grade	195 (2.9) / 6,786	0.67 (0.55, 0.81)	0.64 (0.52, 0.79)	0.73 (0.60, 0.90)
Higher	74 (1.6) / 4,605	0.43 (0.33, 0.57)	0.42 (0.32, 0.55)	0.55 (0.41, 0.72)
HNC/HND	69 (1.9) / 3,730	0.42 (0.32, 0.56)	0.42 (0.32, 0.55)	0.53 (0.40, 0.70)
Degree or higher	90 (1.0) / 8,653	0.28 (0.22, 0.35)	0.27 (0.21, 0.35)	0.39 (0.30, 0.51)
p[1]		*<0.001*	*<0.001*	*<0.001*
Deprivation				
1 Most deprived quintile	269 (4.3) / 6,221	1.00	1.00	1.00
2	194 (3.0) / 6,497	0.66 (0.55, 0.80)	0.67 (0.55, 0.82)	0.76 (0.62, 0.92)
3	138 (1.9) / 7,211	0.42 (0.34, 0.52)	0.42 (0.34, 0.52)	0.51 (0.41, 0.63)
4	102 (1.4) / 7,449	0.30 (0.24, 0.38)	0.31 (0.24, 0.39)	0.40 (0.32, 0.51)
5 Least deprived quintile	66 (1.0) / 6,419	0.22 (0.17, 0.29)	0.22 (0.17, 0.29)	0.31 (0.23, 0.41)
p[1]		*<0.001*	*<0.001*	*<0.001*

Social class				
V (unskilled)	101 (5.0) / 2,029	1.00	1.00	1.00
IV (partly skilled)	193 (3.4) / 5,677	0.66 (0.51, 0.85)	0.64 (0.49, 0.82)	0.66 (0.51, 0.85)
IIIM (skilled manual)	178 (3.0) / 6,039	0.43 (0.33, 0.56)	0.41 (0.31, 0.53)	0.45 (0.34, 0.59)
IIINM (skilled non-manual)	111 (1.5) / 7,226	0.33 (0.25, 0.44)	0.32 (0.24, 0.43)	0.41 (0.31, 0.54)
II (Intermediate)	135 (1.4) / 9,836	0.25 (0.19, 0.33)	0.23 (0.18, 0.31)	0.32 (0.24, 0.42)
I (Professional)	22 (1.3) / 1,750	0.20 (0.13, 0.32)	0.20 (0.13, 0.33)	0.30 (0.19, 0.49)
p[1]		*<0.001*	*<0.001*	*<0.001*
Income				
1 Lowest quintile	142 (3.7) / 3,891	1.00	1.00	1.00
2	113 (2.2) / 5,059	0.58 (0.45, 0.75)	0.59 (0.45, 0.76)	0.64 (0.49, 0.84)
3	97 (1.9) / 5,032	0.47 (0.36, 0.62)	0.45 (0.35, 0.59)	0.54 (0.41, 0.71)
4	62 (1.1) / 5,676	0.25 (0.19, 0.34)	0.24 (0.18, 0.33)	0.31 (0.23, 0.43)
5 Highest quintile	51 (0.9) / 5,938	0.19 (0.14, 0.26)	0.16 (0.12, 0.23)	0.23 (0.16, 0.32)
p[1]		*<0.001*	*<0.001*	*<0.001*

[1]p for linear trend across socioeconomic status categories

Table 3: Odds ratio (95% confidence interval) for alcohol admissions/deaths plus prescriptions according to drinking status by socioeconomic status (sample limited to complete cases)

	Higher socioeconomic position		Lower socioeconomic position	
	n events / Total N	OR (95% CI)[1]	n events / Total N	OR (95% CI)[1]
By highest educational qualification[2]				
Non-drinker	14 / 1,359	1.44 (0.79, 2.61)	49 / 2,866	1.72 (1.14, 2.58)
Light drinker (ref)	50 / 7,216	1.00	127 / 7,558	1.80 (1.29, 2.52)
Moderate drinker	49 / 4,019	1.47 (0.99, 2.19)	103 / 3,094	2.79 (1.97, 3.95)
Heavy drinker	71 / 3,702	2.17 (1.51, 3.14)	166 / 2,643	4.95 (3.56, 6.89)
Excessive drinker	49 / 692	6.77 (4.48, 10.22)	91 / 700	9.69 (6.70, 14.04)
By deprivation[3]				
Non-drinker	28 / 2,094	1.64 (1.05, 2.57)	35 / 2,128	1.71 (1.13, 2.60)
Light drinker (ref)	68 / 9,287	1.00	109 / 5,472	2.30 (1.69, 3.13)
Moderate drinker	56 / 4,680	1.38 (0.97, 1.98)	96 / 2,417	3.65 (2.65, 5.03)
Heavy drinker	95 / 4,187	2.48 (1.80, 3.41)	142 / 2,143	5.90 (4.36, 8.00)
Excessive drinker	59 / 831	6.97 (4.83, 10.07)	81 / 558	11.75 (8.25, 16.73)
By social class[4]				
Non-drinker	18 / 1,805	1.26 (0.74, 2.15)	38 / 2,057	1.72 (1.13, 2.60)
Light drinker (ref)	60 / 8,292	1.00	111 / 5,961	2.00 (1.45, 2.76)
Moderate drinker	50 / 4,199	1.43 (0.97, 2.08)	97 / 2,712	3.16 (2.26, 4.42)
Heavy drinker	91 / 3,808	2.79 (2.00, 3.89)	137 / 2,376	4.73 (3.43, 6.53)
Excessive drinker	49 / 708	7.09 (4.77, 10.54)	89 / 639	11.02 (7.67, 15.82)
By income[5]				

Non-drinker	13 / 1,347	1.26 (0.68, 2.33)	24 / 1,728	1.58 (0.96, 2.59)
Light drinker (ref)	53 / 7,262	1.00	71 / 4,300	1.99 (1.38, 2.86)
Moderate drinker	40 / 3,787	1.28 (0.85, 1.94)	39 / 1,427	2.73 (1.78, 4.18)
Heavy drinker	63 / 3,555	2.04 (1.41, 2.96)	72 / 1,162	6.02 (4.15, 8.71)
Excessive drinker	41 / 695	5.79 (3.78, 8.88)	49 / 333	13.20 (8.64, 20.17)

[1]Adjusted for age, sex, wave, smoking, BMI and binge drinking in last week; [2]None/other school/standard grade (lower socioeconomic status) versus Higher/HNC/HND/Degree or higher (higher socioeconomic status); [3]Most deprived two quintiles versus least deprived three quintiles; [4]Manual versus non-manual occupations; [5]Lowest two quintiles versus highest three quintiles

Web Appendix 9: Assessment of potential selection bias by repeating main analysis for 72% subsample with repeated area-based deprivation measures

Hazard ratio (95% confidence interval) for alcohol admissions/deaths according to socioeconomic position (restricted to those with deprivation measured during follow-up)

	N events / person years	Adjusted for age, sex and data collection wave	Adjusted for age, sex, wave, alcohol consumption and binge drinking	Adjusted for age, sex, wave, alcohol, binge drinking, BMI and smoking status
Highest educational qualification				
Degree or higher	85 / 60,894.3	1.00	1.00	1.00
HNC/HND	82 / 35,679.3	1.63 (1.21, 2.22)	1.42 (0.99, 2.05)	1.28 (0.88, 1.84)
Higher	89 / 39,258.3	1.74 (1.29, 2.35)	1.66 (1.17, 2.36)	1.53 (1.08, 2.16)
Standard grade	296 / 91,181.2	2.56 (2.01, 3.27)	2.33 (1.75, 3.10)	1.93 (1.45, 2.58)
Other school	51 / 16,621.9	2.66 (1.87, 3.80)	2.49 (1.68, 3.73)	1.96 (1.31, 2.95)
None	419 / 103,000.9	3.66 (2.88, 4.65)	3.37 (2.56, 4.44)	2.48 (1.87, 3.28)
p[1]		*<0.001*	*<0.001*	*<0.001*
Deprivation				
5 Least deprived quintile	93 / 59,260.3	1.00	1.00	1.00
4	142 / 68,251.6	1.32 (1.02, 1.72)	1.32 (0.94, 1.83)	1.22 (0.88, 1.70)
3	166 / 71,252.1	1.52 (1.18, 1.96)	1.67 (1.22, 2.29)	1.45 (1.05, 1.99)
2	243 / 73,240.0	2.25 (1.77, 2.86)	2.54 (1.88, 3.43)	2.05 (1.51, 2.78)
1 Most deprived quintile	377 / 74,148.2	3.47 (2.76, 4.35)	3.50 (2.62, 4.68)	2.53 (1.88, 3.41)
p[1]		*<0.001*	*<0.001*	*<0.001*

Social class				
I (Professional)	21 / 14,338.1	1.00	1.00	1.00
II (Intermediate)	153 / 85,220.2	1.50 (0.95, 2.38)	1.29 (0.77, 2.15)	1.18 (0.71, 1.97)
IIINM (skilled non-manual)	132 / 76,034.0	1.91 (1.20, 3.04)	1.52 (0.89, 2.58)	1.30 (0.76, 2.23)
IIIM (skilled manual)	262 / 66,773.2	2.66 (1.71, 4.15)	2.14 (1.29, 3.53)	1.65 (1.00, 2.74)
IV (partly skilled)	255 / 58,332.1	3.94 (2.52, 6.15)	3.37 (2.04, 5.57)	2.42 (1.46, 4.02)
V (unskilled)	129 / 25,507.8	4.98 (3.13, 7.93)	4.16 (2.44, 7.11)	2.95 (1.72, 5.08)
p[1]		*<0.001*	*<0.001*	*<0.001*
Income				
5 Highest quintile	41 / 22,949.7	1.00	1.00	1.00
4	56 / 21,782.5	1.50 (1.00, 2.25)	1.54 (1.02, 2.34)	1.42 (0.93, 2.15)
3	72 / 21,434.1	2.13 (1.45, 3.13)	2.26 (1.52, 3.47)	1.95 (1.30, 2.91)
2	80 / 24,609.7	2.24 (1.52, 3.29)	2.65 (1.78, 3.94)	2.10 (1.41, 3.15)
1 Lowest quintile	110 / 18,545.5	4.00 (2.78, 3.73)	4.31 (2.95, 6.31)	3.23 (2.19, 4.76)
p[1]		*<0.001*	*<0.001*	*<0.001*

[1]p for linear trend across socioeconomic status categories

Web Appendix 10: Robustness analyses to test social selection hypothesis

Table 1: Difference in area-based deprivation quintile between baseline and follow-up (first hospital admission, prescription or death) by drinking status at baseline for respondents interviewed from 2008 onwards

Difference in SIMD quintile	Baseline drinking status<br>Non/ex drinker<br>(N=1,916)	Light/moderate drinker<br>(N=7,285)	Heavy/excessive drinker<br>(N=2,198)
-4 (biggest negative (downward) difference)	1 (0.1)	4 (0.1)	3 (0.1)
-3	2 (0.1)	22 (0.3)	11 (0.5)
-2	11 (0.6)	57 (0.8)	25 (1.1)
-1	51 (2.7)	267 (3.7)	87 (4.0)
0 (no difference)	1,782 (93.0)	6,604 (90.7)	1,963 (89.3)
1	49 (2.6)	208 (2.9)	76 (3.5)
2	11 (0.6)	74 (1.0)	19 (0.9)
3	5 (0.3)	43 (0.6)	10 (0.5)
4 (biggest positive (upward) difference)	4 (0.2)	6 (0.1)	4 (0.2)
Negative difference	65 (3.4)	350 (4.8)	126 (5.7)
No difference	1,782 (93.0)	6,604 (90.7)	1,963 (89.3)
Positive difference	69 (3.6)	331 (4.5)	109 (5.0)
Mean difference relative to non-drinkers			
adjusted for baseline deprivation quintile	0.00	0.02 (-0.00, 0.05)	0.02 (-0.01, 0.05)
adjusted for baseline deprivation quintile, age and sex	0.00	0.02 (-0.01, 0.04)	0.01 (-0.02, 0.04)
adjusted for baseline deprivation quintile, age, sex and time from interview to prescription	0.00	0.01 (-0.01, 0.04)	0.01 (-0.02, 0.04)
adjusted for baseline deprivation quintile, age, sex, time from interview to prescription and wave	0.00	0.01 (-0.01, 0.04)	0.01 (-0.02, 0.04)

Table 2: Difference in area-based deprivation quintile between baseline and follow up (hospital admission, prescription but excluding death) by drinking status at baseline

Difference in SIMD quintile	Baseline drinking status<br>Non/ex drinker (N=2,753)	Light/moderate drinker (N=15,853)	Heavy/excessive drinker (N=4,700)
-4 (biggest negative (downward) difference)	8 (0.3)	43 (0.3)	16 (0.3)
-3	13 (0.5)	135 (0.9)	41 (0.9)
-2	33 (1.2)	301 (1.9)	121 (2.6)
-1	92 (3.3)	670 (4.2)	203 (4.3)
0 (no difference)	2,451 (89.0)	13,532 (85.4)	3,888 (82.7)
1	103 (3.7)	628 (4.0)	235 (5.0)
2	34 (1.2)	333 (2.1)	117 (2.5)
3	13 (0.5)	176 (1.1)	60 (1.3)
4 (biggest positive (upward) difference)	6 (0.2)	35 (0.2)	19 (0.4)
Negative difference	146 (5.3)	1,149 (7.2)	381 (8.1)
No difference	2,451 (89.0)	13,532 (85.4)	3,888 (82.7)
Positive difference	156 (5.7)	1,172 (7.4)	431 (9.2)
Mean difference relative to non-drinkers			
adjusted for baseline deprivation quintile	0.00	0.05 (0.03, 0.08)	0.08 (0.04, 0.11)
adjusted for baseline deprivation quintile, age and sex	0.00	0.05 (0.02, 0.08)	0.07 (0.04, 0.10)
adjusted for baseline deprivation quintile, age, sex and time from interview to prescription	0.00	0.05 (0.02, 0.08)	0.07 (0.04, 0.10)
adjusted for baseline deprivation quintile, age, sex, time from interview to prescription and wave	0.00	0.05 (0.02, 0.08)	0.07 (0.04, 0.10)

Table 3: Difference in area-based deprivation quintile between baseline and follow up (hospital admission, prescription but excluding death) by drinking status at baseline for participants from 2008 onwards

Difference in SIMD quintile	Non/ex drinker (N=1,594)	Baseline drinking status<br>Light/moderate drinker (N=6,676)	Heavy/excessive drinker (N=2,060)
-4 (biggest negative (downward) difference)	1 (0.1)	4 (0.1)	3 (0.2)
-3	2 (0.1)	22 (0.3)	10 (0.5)
-2	8 (0.5)	56 (0.8)	25 (1.2)
-1	45 (2.8)	253 (3.8)	83 (4.0)
0 (no difference)	1,476 (92.6)	6,022 (90.2)	1,838 (89.2)
1	45 (2.8)	200 (3.0)	70 (3.4)
2	10 (0.6)	73 (1.1)	18 (0.9)
3	5 (0.3)	41 (0.6)	9 (0.4)
4 (biggest positive (upward) difference)	2 (0.1)	5 (0.1)	4 (0.2)
Negative difference	56 (3.5)	335 (5.0)	121 (5.9)
No difference	1,476 (92.6)	6,022 (90.2)	1,838 (89.2)
Positive difference	62 (3.9)	319 (4.8)	101 (4.9)
Mean difference relative to non-drinkers			
adjusted for baseline deprivation quintile	0.00	0.02 (-0.00, 0.05)	0.01 (-0.02, 0.05)
adjusted for baseline deprivation quintile, age and sex	0.00	0.02 (-0.01, 0.04)	0.00 (-0.03, 0.04)
adjusted for baseline deprivation quintile, age, sex and time from interview to prescription	0.00	0.02 (-0.01, 0.04)	0.00 (-0.03, 0.04)
adjusted for baseline deprivation quintile, age, sex, time from interview to prescription and wave	0.00	0.02 (-0.01, 0.04)	0.00 (-0.03, 0.04)

Table 4: Difference in SIMD quintile between baseline and follow up (hospital admission, prescription but excluding death) by drinking status at baseline

Difference in SIMD quintile	Baseline drinking status<br>Non/ex drinker<br>(N=2,753)	 Non binge drinker<br>(N=11,326)	 Binge drinker<br>(N=2,957)
-4 (biggest negative (downward) difference)	8 (0.3)	24 (0.2)	5 (0.2)
-3	13 (0.5)	70 (0.6)	28 (1.0)
-2	33 (1.2)	179 (1.6)	69 (2.3)
-1	92 (3.3)	441 (3.9)	131 (4.4)
0 (no difference)	2,451 (89.0)	9,980 (88.1)	2,441 (82.6)
1	103 (3.7)	366 (3.2)	154 (5.2)
2	34 (1.2)	160 (1.4)	82 (2.8)
3	13 (0.5)	87 (0.8)	36 (1.2)
4 (biggest positive (upward) difference)	6 (0.2)	19 (0.2)	11 (0.4)
Negative difference	146 (5.3)	714 (6.3)	233 (7.9)
No difference	2,451 (89.0)	9,980 (88.1)	2,441 (82.6)
Positive difference	156 (5.7)	632 (5.6)	283 (9.6)
Mean difference relative to non-drinkers			
adjusted for baseline deprivation quintile	0.00	0.04 (0.02, 0.07)	0.06 (0.03, 0.09)
adjusted for baseline deprivation quintile, age and sex	0.00	0.04 (0.02, 0.07)	0.06 (0.02, 0.09)
adjusted for baseline deprivation quintile, age, sex and time from interview to prescription	0.00	0.04 (0.02, 0.07)	0.06 (0.02, 0.09)
adjusted for baseline deprivation quintile, age, sex, time from interview to prescription and wave	0.00	0.04 (0.01, 0.07)	0.05 (0.02, 0.09)

Table 5: Difference in area-based deprivation quintile between baseline and follow up (hospital admission, prescription but excluding death) by drinking status at baseline for participants from 2008 onwards

Difference in SIMD quintile	Baseline drinking status<br>Non/ex drinker<br>(N=1,594)	 Non binge drinker<br>(N=7,105)	 Binge drinker<br>(N=1,625)
-4 (biggest negative (downward) difference)	1 (0.1)	5 (0.1)	1 (0.1)
-3	2 (0.1)	21 (0.3)	10 (0.6)
-2	8 (0.5)	63 (0.9)	19 (1.2)
-1	45 (2.8)	279 (3.9)	58 (3.6)
0 (no difference)	1,476 (92.6)	6,426 (90.4)	1,428 (87.9)
1	45 (2.8)	202 (2.8)	69 (4.3)
2	10 (0.6)	62 (0.9)	29 (1.8)
3	5 (0.3)	41 (0.6)	8 (0.5)
4 (biggest positive (upward) difference)	2 (0.1)	6 (0.1)	3 (0.2)
Negative difference	56 (3.5)	338 (4.8)	88 (5.4)
No difference	1,476 (92.6)	6,426 (90.4)	1,428 (87.9)
Positive difference	62 (3.9)	311 (4.4)	109 (6.7)
Mean difference relative to non-drinkers			
adjusted for baseline deprivation quintile	0.00	0.02 (-0.01, 0.04)	0.03 (-0.00, 0.07)
adjusted for baseline deprivation quintile, age and sex	0.00	0.01 (-0.01, 0.04)	0.02 (-0.01, 0.06)
adjusted for baseline deprivation quintile, age, sex and time from interview to prescription	0.00	001 (-0.01, 0.04)	0.02 (-0.02, 0.06)
adjusted for baseline deprivation quintile, age, sex, time from interview to prescription and wave	0.00	0.01 (-0.01, 0.04)	0.02 (-0.02, 0.05)

Table 6: Difference in area-based deprivation quintile between baseline and follow-up (from first hospital admission, prescription or death), distinguishing ex-drinkers from non-drinkers

Difference in area-based deprivation quintile	Baseline drinking status			
	Non-drinker (N=1,781)	Ex-drinker (N=1,848)	Light/moderate drinker (N=18,439)	Heavy/excessive drinker (N=5,351)
-4 (biggest negative (downward) difference)	6 (0.3)	3 (0.2)	46 (0.3)	16 (0.3)
-3	9 (0.5)	5 (0.3)	144 (0.8)	45 (0.8)
-2	23 (1.3)	18 (1.0)	326 (1.8)	125 (2.3)
-1	52 (2.9)	59 (3.2)	733 (4.0)	214 (4.0)
0 (no difference)	1,583 (88.9)	1,687 (91.3)	15,915 (86.3)	4,492 (84.0)
1	67 (3.8)	51 (2.8)	692 (3.8)	251 (4.7)
2	28 (1.6)	13 (0.7)	352 (1.9)	123 (2.3)
3	8 (0.5)	8 (0.4)	193 (1.1)	65 (1.2)
4 (biggest positive (upward) difference)	5 (0.3)	4 (0.2)	38 (0.2)	20 (0.4)
Negative difference	90 (5.1)	85 (4.6)	1,249 (6.8)	400 (7.5)
No difference	1,583 (88.9)	1,687 (91.3)	15,915 (86.3)	4,492 (84.0)
Positive difference	108 (6.1)	76 (4.1)	1,275 (6.9)	459 (8.6)
Mean difference relative to non-drinkers				
adjusted for baseline deprivation quintile	0.00	-0.03 (-0.08, 0.01)	0.02 (-0.00, 0.06)	0.05 (0.02, 0.09)
adjusted for baseline deprivation quintile, age and sex	0.00	-0.03 (-0.08, 0.01)	0.03 (-0.01, 0.06)	0.05 (0.01, 0.08)
adjusted for baseline deprivation quintile, age, sex and time from interview to prescription	0.00	-0.03 (-0.08, 0.01)	0.03 (-0.01, 0.06)	0.05 (0.01, 0.08)
adjusted for baseline deprivation quintile, age, sex, time from interview to prescription and wave	0.00	-0.03 (-0.08, 0.01)	0.03 (-0.01, 0.06)	0.04 (0.01, 0.08)

Table 7: Difference in area-based deprivation quintile between baseline and follow-up (from first hospital admission, prescription or death), distinguishing excessive drinkers from heavy drinkers

	Baseline drinking status			
Difference in area-based deprivation quintile	Non/ex drinker (N=3,629)	Light/moderate drinker (N=18,439)	Heavy drinker (N=4,332)	Excessive drinker (N=1,019)
-4 (biggest negative (downward) difference)	9 (0.3)	46 (0.3)	14 (0.3)	2 (0.2)
-3	14 (0.4)	144 (0.8)	32 (0.7)	13 (1.3)
-2	41 (1.1)	326 (1.8)	100 (2.3)	25 (2.5)
-1	111 (3.1)	733 (4.0)	175 (4.0)	39 (3.8)
0 (no difference)	3,270 (90.1)	15,915 (86.3)	3,627 (83.7)	865 (84.9)
1	118 (3.3)	692 (3.8)	203 (4.7)	48 (4.7)
2	41 (1.1)	352 (1.9)	114 (2.6)	9 (0.9)
3	16 (0.4)	193 (1.1)	50 (1.2)	15 (1.5)
4 (biggest positive (upward) difference)	9 (0.3)	38 (0.2)	17 (0.4)	3 (0.3)
Negative difference	175 (4.8)	1,249 (6.8)	321 (7.4)	79 (7.8)
No difference	3,270 (90.1)	15,915 (86.3)	3,627 (83.7)	865 (84.9)
Positive difference	184 (5.1)	1,275 (6.9)	384 (8.9)	75 (7.4)
Mean difference relative to non-drinkers				
adjusted for baseline deprivation quintile	0.00	0.05 (0.02, 0.07)	0.08 (0.06, 0.11)	0.01 (-0.04, 0.05)
adjusted for baseline deprivation quintile, age and sex	0.00	0.04 (0.02, 0.07)	0.08 (0.05, 0.11)	0.00 (-0.05, 0.05)
adjusted for baseline deprivation quintile, age, sex and time from interview to prescription	0.00	0.04 (0.02, 0.07)	0.08 (0.05, 0.11)	-0.00 (-0.05, 0.05)
adjusted for baseline deprivation quintile, age, sex, time from interview to prescription and wave	0.00	0.04 (0.02, 0.07)	0.08 (0.05, 0.11)	-0.00 (-0.05, 0.04)
